# Enlargements of the Spleen—VII

**Published:** 1908-09-19

**Authors:** 


### ENLARGEMENTS OF THE SPLEEN—VII.

### SPLENIC ENLARGEMENTS WITH DISTINCTIVE BLOOD CHANGES.—*Continued.*

5. *Malaria, Ague, and Kala-azar.*—Ague and malaria are practically identical, but from the point of view of the spleen itself a distinction needs to be drawn between them; for the spleen of actual malaria can be diagnosed by blood examination, whereas the ague-cake spleen of former malaria outlasts the positive blood changes, and blood examination alone will not suffice in its diagnosis.

Kala-azar is here mentioned almost in the same breath with malaria, because it has only recently been differentiated from the latter; it is now known to be a very different disease, and it is important to recognise this, because many patients who return from certain parts of India, particularly from the region of Assam, may suffer from pyrexial attacks which are attributed to "malaria," the spleen may be enlarged, and yet when the blood is examined no malarial parasites are found. Kala-azar is not due to the plasmodium malariæ, and no parasites are found in the peripheral blood in these cases. The disease seems to be caused by an entirely different protozoon related to the trypanosomes, and the diagnosis is made by the discovery of Leishman-Donovan bodies not in blood films, but in films made from the material obtained upon diagnostic puncture of the spleen itself. It is more than likely that many cases of splenic enlargement in patients returned from the tropics and regarded as suffering from malaria may really have been due to Kala-azar—hence the non-appearance of malarial parasites in films of the patients' blood.

Returning now to the ague-cake spleen, this can only be diagnosed with certainty when the patient has been known to have had repeated attacks of malaria, as proved by blood-films at that time. There are no specific blood changes associated with the ague-cake spleen itself. The parasites have gone, unless there should be recrudescence of the original malaria. Ague-cake spleens used to be fairly common in the English Fens and in other swampy districts in Great Britain. Since these have been drained English ague has become much rarer, and most ague-cake spleens are found in those who have made long sojourns in other parts of Europe or in the tropics. After repeated attacks of malaria and long exposure to malarial infection a condition may be produced which is characterised by a considerable or enormous enlargement of the spleen, grave anæmia, an earthy, sallow complexion, loss of flesh, hæmorrhages, lassitude, attacks of dizziness, loss of appetite, frontal headaches, and pains in the limbs. The spleen becomes very hard, may weigh 10 lb. or more, and be tender. It can only be distinguished from spleno-medullary leuchæmia by careful blood examination. In post-malarial cachexia the red corpuscles are much reduced in number—they may fall as low as 2,000,000 per cubic millimetre, or 1,000,000, or even less. There may be poikilocytosis and polychromatosis, and nucleated red corpuscles may be present. The hæmoglobin is even more diminished than are the red cells, so that the colour index is low. The white cells, however, show little characteristic change from normal.

In the early stages of the actual malarial infection the spleen becomes enlarged and soft from hyperæmia. The different kinds and types of malarial attacks need not be gone into here. Suffice it to say that during the paroxysm the spleen can often be felt projecting below the costal margin and presenting a soft, indefinite lower border. If the patient has been the subject of malaria before, and already has the beginning of an ague-cake spleen, even the latter will be found temporarily to enlarge further during the attack. The most conclusive evidence as to the nature of the disease is obtained by a careful blood examination. One point is too often forgotten when films from a malarial patient are sent to a laboratory, and that is that the parasites very quickly disappear from the peripheral blood when quinine has been administered. The films should be made at once, before quinine is given, and preferably just at the time when an attack is due, for it is then that the hæmatozoa are at that stage of development when they are most easily stained and detected.

If, however, quinine has been given so that it is hopeless to expect to find the parasites in the films, there are two other points that are worth knowing about the blood in malaria, for although they cannot actually clinch the diagnosis they can corroborate it very substantially. These two points are: first, that there is no leucocytosis in malaria; secondly, that there is a marked relative increase in the large hyaline lymphocytes.

6. *Typhoid Fever.*—The chief diagnostic point about the blood in this disease is Widal's reaction; in no other disease will the serum diluted 200 times cause clumping of typhoid bacilli in half an hour. The difficulty is that Widal's reaction is seldom positive until after the end of the first week. But the blood affords another means of corroborating the diagnosis depending upon the facts: first, that in typhoid fever, there is no leucocytosis; secondly, that there is nearly always a relative diminution in the polymorphonuclear cells in the differential leucocyte count, with a corresponding relative increase in the small lymphocytes.

